# Superporous acrylic acid and HPMC hydrogels of mefenamic acid: Formulation, characterization and optimization by central composite design

**DOI:** 10.3389/fbioe.2022.1057627

**Published:** 2022-12-15

**Authors:** Hafeez Ullah Khan, Samar Aziz, Safirah Maheen, Ikramullah Khan, Mehwish Andleeb, Hina Younis, Sajjad Haider, Adnan Haider, Muhammad Saeed Akhtar, Syed Salman Shafqat

**Affiliations:** ^1^ Department of Pharmaceutics, University of Sargodha, Sargodha, Pakistan; ^2^ Department of Pharmaceutics, Faculty of Pharmaceutical Sciences, Government College University Faisalabad, Faisalabad, Pakistan; ^3^ Department of Chemical Engineering, College of Engineering, King Saud University, Riyadh, Saudi Arabia; ^4^ Department of Biological Sciences, National University of Medical Sciences, Rawalpindi, Pakistan; ^5^ School of Chemical Engineering, Yeungnam University, Gyeongsan, South Korea; ^6^ Department of Chemistry, Division of Science and Technology, University of Education, Lahore, Pakistan

**Keywords:** acrylic acid, analgesic, central composite rotatable design, gas blowing method, HPMC, mefenamic acid, superporous hydrogels

## Abstract

The purpose of the study was to devise the superporous hydrogels (SPHs) of mefenamic acid (MA) to acquire the sustained action of the MA in the body. The superporous hydrogels of mefenamic acid were formulated by employing the gas blowing method. The central composite rotatable design (CCRD) was applied to optimize the effect of independent formulation factors like acrylic acid (AC), HPMC and glycerol (GLY) over dependent variables like porosity, viscosity, drug content and swelling ratio of superporous hydrogels in water, phosphate buffer (pH 6.8) and in 0.1N HCl (pH 1.2). A number of characteristics such as void fraction, surface morphology by Scanning electron microscopy (SEM) and *in vitro* drug release study were governed along with physico-chemical analysis by Fourier transform infrared spectroscopy (FTIR), Differential scanning calorimetry (DSC) and appraised statistically by employing the ANOVA. The comparative analgesic activity of optimized superporous hydrogel formulation SPH17 was also analyzed by using tail flick method. The Fourier transform infrared spectroscopy and Differential scanning calorimetry studies approved the physical compatibility between the polymers and the drug. The Scanning electron microscopy study specified micrographic insight about the structure of formed formulations comprising presence of pores, fibers and drug-hole aggregates. The superporous hydrogels were detected to be low dense as they expressed density lower than 0.75 g/cc. The decrease in concentration of the polymers and cross linker contributed towards the increase in the void fraction of the superporous hydrogel formulations. The optimized formulation SPH 17 exhibited a highly sustained release of MA for up to 10 h in the both 0.1 N HCl and phosphate buffer (66.6%) media. The non-fickian release of drug revealed the coupling of the diffusion and polymer relaxation mechanism of the drug release from the formulations. The obtained outcomes suggested that analgesic effect of SPH 17 was significantly (*p* < 0.05) higher than that of simple suspension of mefenamic acid and total analgesic effect duration for superporous hydrogel was also doubled as compared to the duration of analgesic effect produced by drug suspension. The successfully formulated SPH with HPMC K100M as a gelling agent had sustained the action of the mefenamic acid (MF) by improving its poor solubility and permeability. The introduction of inter-penetrating polymeric network (acrylic acid) using glycerol as a cross linker impart increased residence time to superporous hydrogels which ultimately enhanced the feasibility of using superporous hydrogel as oral sustained release devices particularly for gastric retention.

## Introduction

The safe and effective way of drug delivery is a very critical parameter to be considered for patients. For drug delivery, polymers play a vital role in modifying drug release (Lv et al., 2021) and among these, highly porous (Tang et al., 2022) hypercrosslinked polymers (HCPs) (Song et al., 2022) have been found very useful for wide variety of drug delivery systems. The morphology, porosity and bio-responsiveness of polymers have significant impact on sustaining or controlling the drug release from the drug delivery system (Tang et al., 2022). Now a days, electrospinning technology is gaining a wide spread acceptance in drug delivery, where the ingredients are loaded or coated in firstly electrospun nanofibers as reported for loading of antiviral drug acyclovir on electrospun. Polyacrylonitrile as the filament-forming polymer and a trilayer nanodepot where the acyclovir was loaded matrix in highly porous cellulose acetate (Wang et al., 2020).

Superporous hydrogels (SPHs) are an innovative type of superabsorbent polymers that are primarily described by fast swelling, high porosity and large swelling ratio with a 3D network of hydrophilic polymers (Polnok et al., 2004) but having a drawback of poor mechanical strength. However, mechanical strength can ominously be improved by creating interpenetrating polymeric networks by adding cross-linked polymers. A variety of natural and synthetic polymers including chitosan, chitin, alginates, poly (acrylic acid) and poly (Vinylpyrrolidone) have potential to boost the residence time of delivery system in the intestinal tract for at least 1 h owing to their chemical fixation properties, and made these systems secure in usage (Nagpal et al., 2013; Zakerikhoob et al., 2021). Numerous colloidal delivery systems such as liposomes, solid lipid nanoparticles (SLNs), nanostructured lipid carriers (NLCs) and micro emulsions have been commercialized to recuperate bioavailability and to extend the residence time of a drug (Shastri et al., 2010a; Gan et al., 2013).

Superporous hydrogels can be designed by either polymerization of hydrophilic monomers in the manifestation of gas blowing agent, which diminishes the polymer substance immunogenicity and rises the enzymatic degradation confrontation (Figueiro et al., 2006; Zhang et al., 2011). The SPHs can also be designed by simple cross linking prevailing the hydrophilic polymer chains (Md et al., 2022). Superporous hydrogels of drugs such as octreotide, carvedilol, amoxicillin, desmopressin, rosiglitazone maleate were designed by means of polymers such as chitosan, xanthan gum, cellulose derivatives, polyacrylic acid, carbopol, and poloxamer by integrating the drug *via* diverse methods in hydrogel drug delivery system (Gannu et al., 2009; Ghica et al., 2011; Gonzalez-Mira et al., 2012; Ramteke and Nath, 2012; Bhalla, Nagpal; Jafari et al., 2019; Khorasani et al., 2021). Hydroxypropyl methylcellulose (HPMC) is the utmost imperative hydrophilic, biodegradable, biocompatible, non-toxic, low cost carrier material exploited in the articulation of hydrogel for drug delivery (Sannino et al., 2009) where it hydrates to develop a gelatinous layer which regulates the transport of water in the system and diffusion of drug out of the system by polymer chain relaxation with volume expansion (Gafourian et al., 2007).

Acrylic acid (AC) is a pH and electrically sensitive, bio adhesive, biocompatible and antibacterial material owing to its trailing carboxylic groups, bring forth slight antigenic reaction in the *in vivo* environment and reveal high tolerance. Polyacrylic acid (PAA) and its copolymers have been bring into play as a vehicle in drug delivery systems, and in pharmaceutical processes owing to their pH dependent swelling behavior for the sustained release of drugs in ocular, nasal, buccal, gastro-intestinal, epidermal and transdermal drug delivery system (Dimitrov et al., 2003; Huang et al., 2007; Ray et al., 2008). Mefenamic acid is a vastly nominal anti-inflammatory, analgesic and antipyretic drug. It is a water insoluble drug and has an elimination half-life of approximately 2 h. For that reason, it is an appropriate candidate for the design of sustained release bio adhesive drug delivery systems for having a consistent prolonged therapeutic response.

The current study was devised to design mefenamic acid loaded superporous hydrogel drug delivery system for bettering its water solubility and prolonging its half-life and to study the influence of formulation variables on different properties of the hydrogels (drug release) in order to optimize the formulations by a statistical procedure-central composite design-alongwith physicochemical characterization of superporous hydrogels. Current research focused on communicating an innovative acrylic acid/hydroxypropyl methylcellulose (AC/HPMC) superporous hydrogels that were prepared by gas blowing method with the assistance of a crosslinking agent, glycerol. In this respect, exploration of numerous samples of superporous hydrogels was carried out by means of altering polymeric, monomeric compositions and degree of crosslinking. Release of the model drug, Mefenamic acid was studied in USP phosphate buffer and 0.1N HCl from the AC/HPMC hydrogels. Superporous hydrogels structure was inspected and distinguished by SEM. Determination of *in vitro* gelling capacity, void fraction examination and *in vivo* analgesic evaluation of superporous hydrogels were also accomplished successfully.

## Materials and methods

### Materials

Hydroxypropyl methylcellulose (HPMC K100M), benzoyl peroxide and xanthan gum were acquired as gift samples from Wilshire Pharmaceuticals, Pakistan, Derma Techno Pharma, Pakistan and Merck laboratories respectively. Acrylic acid (AC), glycerol (GLY), Tween 80 and sodium bicarbonate (NaHCO_3_) were purchased from Sigma Aldrich. Mefenamic acid (MF) was received as a gift sample from Qintar Pharmaceutical, Sargodha, Pakistan. All of the chemicals used in the preparation of SPHs were of analytical grade.

### Synthesis of superporous hydrogels

Formulation of SPHs was accomplished by gas blowing method. First of all, the stock solutions of acrylic acid, HPMC and glycerol were prepared for SPHs formulations (SPH 1-SPH 17) corresponding to the concentrations as presented in the Table 1. Then, the stock solutions of xanthan gum and Tween 80 were prepared and their concentration and volume used for SPHs formulations (SPH 1-SPH 17) are presented in Table 2. The concentration and amount of initiator (benzoyl peroxide) along with the amount of sodium bicarbonate (NaHCO_3_), water and mefenamic acid that was used in the preparation of seventeen formulations of SPHs was presented in the Table 2. The seventeen formulations of SPHs were designed in a beaker by adding and dissolving acrylic acid, HPMC, mefenamic acid, and xanthan gum, cross linking agent (glycerol), tween 80, and distilled water respectively with gentle heating. NaOH (2M) solution was added to adjust pH at 5. Then, NaHCO_3_ was blended with mixture in a rapid way for 10s and mixture was stirred for 10 min constantly by using hot pl; ate magnetic stirrer till the formation of SPHs completed. The SPHs designed for formulations (SPH1-SPH17) were permitted to dry at 50°C for 72 h in a hot air oven. Grinding and sieving was carried out to attain uniform sized particles and stored in airtight container along with desiccator (silica gel) until further use (Chavda et al., 2013).

**TABLE 1:** Coded central composite design for three factors.

Formulations	Coded level of variables			Actual level of variables		
	X1: Acrylic acid (%)	X2: HPMC (%)	X3: Glycerol (%)	X1: Acrylic acid (%)	X2: HPMC (%)	X3: Glycerol (%)
Factorial points						
1	−1	−1	−1	2.20	1.20	0.80
2	1	−1	−1	2.80	1.20	0.80
3	−1	1	−1	2.20	1.80	0.80
4	1	1	−1	2.80	1.80	0.80
5	−1	−1	1	2.20	1.20	2.50
6	1	−1	1	2.80	1.20	2.50
7	−1	1	1	2.20	1.80	2.50
8	1	1	1	2.80	1.80	2.50
Axial points						
9	−1.682	0	0	2.00	1.50	1.65
10	1.682	0	0	3.00	1.50	1.65
11	0	−1.682	0	2.50	1.00	1.65
12	0	1.682	0	2.50	2.00	1.65
13	0	0	−1.682	2.50	1.50	0.22
14	0	0	1.682	2.50	1.50	3.08
Centre points						
15	0	0	0	2.50	1.50	1.65
16	0	0	0	2.50	1.50	1.65
17	0	0	0	2.50	1.50	1.65

**TABLE 2:** Composition of ingredients of central composite batches.

Formulation	Xanthan gum (1%w/v) (ml)	Tween 80 (10% w/v) (ml)	Benzoyl peroxide (50% w/v) (g)	Distilled water (ml)	Sodium bicarbonate (mg)	Drug (mg)
SPH 1	3	2.5	4	2	120	500
SPH 2	3	2.5	4	2	120	500
SPH 3	3	2.5	4	2	120	500
SPH 4	3	2.5	4	2	120	500
SPH 5	3	2.5	4	2	120	500
SPH 6	3	2.5	4	2	120	500
SPH 7	3	2.5	4	2	120	500
SPH 8	3	2.5	4	2	120	500
SPH 9	3	2.5	4	2	120	500
SPH 10	3	2.5	4	2	120	500
SPH 11	3	2.5	4	2	120	500
SPH 12	3	2.5	4	2	120	500
SPH 13	3	2.5	4	2	120	500
SPH 14	3	2.5	4	2	120	500
SPH 15	3	2.5	4	2	120	500
SPH 16	3	2.5	4	2	120	500
SPH 17	3	2.5	4	2	120	500

**SCHEME 1:**
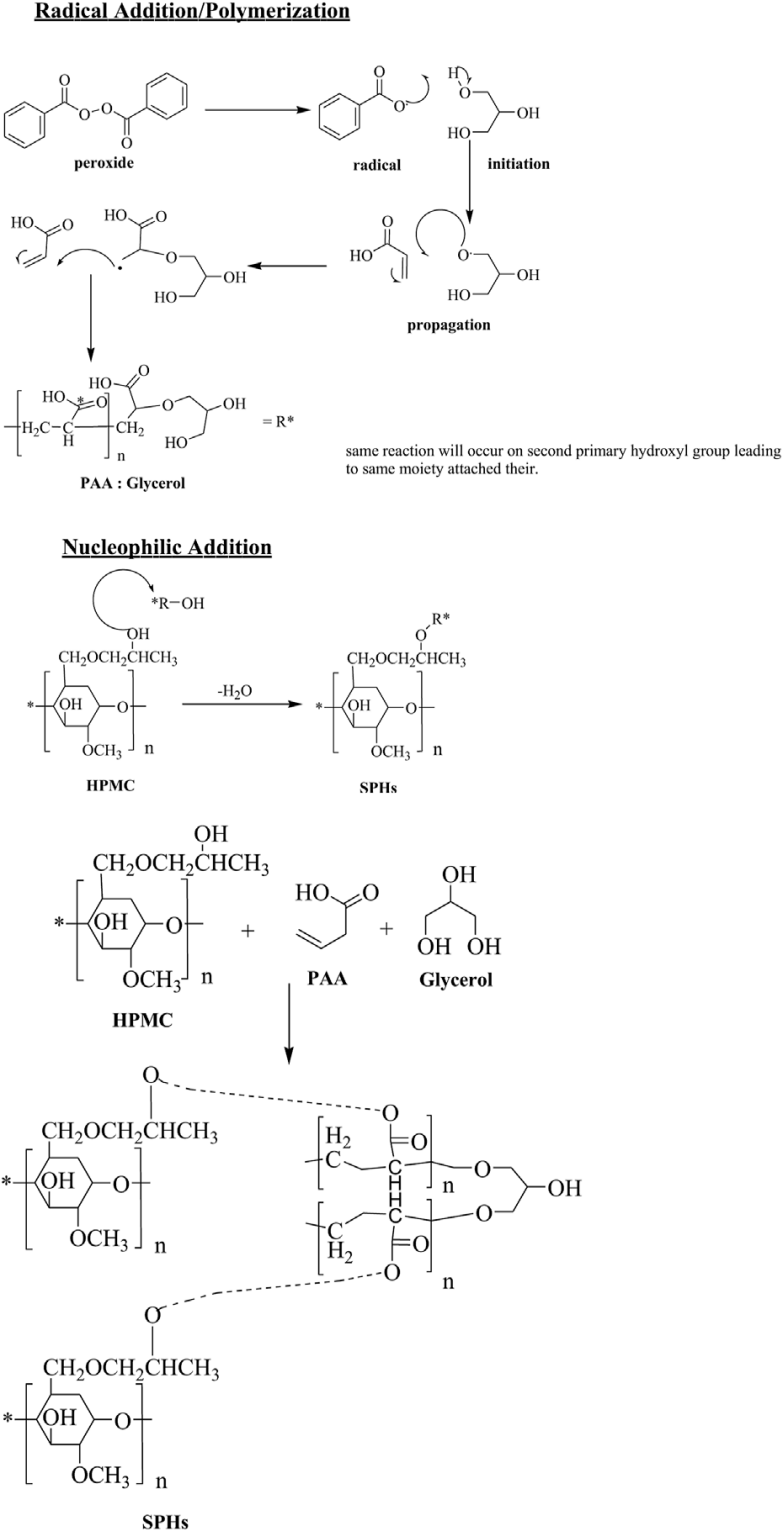
Scheme for the preparation of Acrylic acid and HPMC cross linked superporous hydrogels.

The probable structure and mechanism of formulated gel is revealed in Scheme 1. HPMC is a polymer frequently used in drugs delivery systems due to its thickness and water retention ability. Both of these properties can be imperiled to presence of hydrogen bonding. Presence of higher no of OH groups involved in H-bonding tends to increase viscosity of solution relatively. The free hydroxyl groups also acts as nucleophile for linking with other monomers. Acrylic acid is liquid at room temperature. It contain a carboxylic group and an unsaturated C=C, both of these groups are ample reactive in nature. The COOH groups helps in dissolution in water. The acrylic acid readily polymerizes in the presence of light, heat and peroxides. The C=C can undergo radical initiated addition. On the other hand, nucleophile addition or elimination at carboxyl functional also occurs designating it appropriate for task. Glycerol is a triol. It contains 2-primary and one-secondary hydroxyl group. The primary OH groups are chemically equal and react simultaneously under same conditions. The free rotation of C-C bonds makes special arrangement of attached moieties as far as possible to reduce stearic hindrance.

### Experimental design

Traditionally, pharmaceutical preparations were instigated by varying the one variable at one time but this is a time exhausting procedure, needs a great deal of inventive attempts and complicated one for the determination of the ideal formulation. The utilization of such classic technique is not considered due to non-involvement of combination effect of this technique. Therefore, it is necessary to determine the complication of the pharmaceutical preparations by utilizing the proved statistical tools like central composite design.

In this study, a five-level, three-factor central composite rotatable design (CCRD) was used, utilizing 17 experiments that entailed eight factorial points, six axial points and three central points. The design was elected for its efficient furnishing of enough degree of freedom to settle down the interactions between factors and the main effects. The amount of acrylic acid (2%–3%) as X_1_, amount of HPMC (1%–2%) as X_2_ and that of glycerol (0.3%–3%) as X_3_ was designated as independent variables that were governed in the preliminary studies. The responses such as porosity as Y_1_, viscosity as Y_2_, drug content as Y_3_, swelling ratio in water as Y_4_, swelling ratio in phosphate buffer (pH 6.8) as Y_5_ and swelling ratio in 0.1N HCl (pH 1.2) as Y_6_ were designated as dependent variables. The experimental design was fabricated for the modeling and calculation of the response surface and the statistical evaluation by the application of state ease design expert software (version 11.0). The data acquired for the dependent (response) variables was tailored by the second order model as quadratic polynomial equation;

$$Y=\beta \overset{3}{\underset{i=1}{ko}}+\sum \beta \overset{3}{\underset{i=1}{k_{i}x_{i}}}+\sum \beta \overset{3}{\underset{i=1}{k_{ii}}}\overset{3}{\underset{i=j+1}{x_{i}^{2}}}+\sum \sum \beta k_{ij}x_{i}x_{j}$$ (1)

Where Y is the measured response variable for each factor level to be modeled; βko, βk_i,_ βk_ii,_ and βk_ij_ are constant regression coefficients designating the intercept, linear, quadratic and interaction terms respectively and xi and xj signify the independent variables in coded form. The least square method was employed for the determination of the statistical significance of coefficients of the response function. The evaluation of the goodness of fit of applied model was carried out by determining the *R* ^*2*^ and response surface and contour plots were taken by the utilization of the Design expert (version 11.0).

## Characterization of superporous Hydrogels`

### Porosity measurement of SPH particles

Porosity of SPH particles was determined by submerging the certain quantity of SPH particles in absolute ethanol for a specified time. SPH particles were reweighed after taking out the surplus solvent and porosity was calculated from the following formula (Chavda et al., 2013);

$$Porosity=\frac{\left(w-W_{0}\right)}{ῥV_{T}}$$ (2)

Where, the weight of SPHs before and after immersion in solvent was denoted by “Wo” and “W” respectively while is representing density (0.789) of the solvent and “V” is the volume of SPHs.

### Viscosity

In order to record the viscosity of SPHs, Brookfield viscometer (DV-E viscometer) was utilized in which the formulation was dipped and rotated at 100 rpm at room temperature (Sabale and Vora, 2012).

### Determination of drug content

A specific quatity of SPH (6 mg) was dissolved in absolute ethanol (10 ml), stirred for a time period of 30 min and then filtered through membrane filter (0.45 µm). The sample was taken from filterate and diluted to determine the drug content in the sample by using UV-Visible Spectrophotometer at *λ*max (353 nm) (Sabale and Vora, 2012).

### Swelling of SPH microparticles

The prepared SPH samples (0.10 g) were placed in a series of graduated cylinders (25 ml) encompassing distilled water or Phosphate buffer (pH 6.8) (25 ml) or 0.1 N HCl (pH 1.2) (25 ml), blended and left to stand at 37°C while the volumes of the swollen samples was assessed after 20 min at equilibrium and the following equation was used to calculate swelling value (El-Said et al., 2016):

Swelling Value=Volume of SampleWeight of dry sample (3)

### Density measurement of SPH particles

SPH particles were imperiled to apparent density measurement by means of solvent displacement methods in which predetermined volume of absolute ethanol was catch on to graduated cylinder. SPH particles of identified mass were engrossed in it and displace volume of absolute ethanol by SPH particles was measured.

The apparent density was calculated using following formula (Ibrahim et al., 2013; El-Said et al., 2016);

Density=MV (4)

Where “V” is volume of ethanol displaced by SPH particles and “M” is the mass of SPH.

### pH measurement

For the determination of pH of the SPH samples, digital pH meter (Cole-parmer instrument Co., United States) was utilized in which the probe of the pH meter was dipped into contact with the sample to measure the pH of the SPHs (Ibrahim et al., 2013; Fard et al., 2022).

### Determination of void fraction

Superporous hydrogel samples were dissolved in HCl of pH 1.2 until equilibrium swelling point was attained (Desu et al., 2020) and Void fraction was calculated from following formula;

Void Fraction=Dimension volume of hydrogen/total volume of pores (5)

Where, Dimensions of swollen superporous hydrogel samples define the dimensional volume of hydrogels while total volume of the pores was calculated by subtracting the weight of the dried hydrogel from the weight of the swollen hydrogel (Bhalla, Nagpal).

### *In Vitro* gelling capacity study

For determining the gelling capacity, a SPH sample was retained in a beaker comprising freshly prepared phosphate buffer (pH 6.8) and 0.1 N HCl (pH 1.2) of 100 ml equilibrated at 37°C. Gel formation was governed by visual assessment while time was taken as an evaluation parameter that was requisite for the gelation and for the formed gel to dissolve (Khorasani et al., 2021).

### *In Vitro* drug release studies

*In vitro* drug release study from prepared SPHs of mefenamic acid was accomplished by USP dissolution apparatus, type II in 500 ml of 0.1 N HCl at 37 ± 0.5°C for 10 h by rotating the paddles at 50 rpm. The 5 ml aliquots were withdrawn, replaced, filtered and assayed by using UV-visible Sspectrophotometer at *λ*max (353 nm) and same procedure was performed in the phosphate buffer (pH 6.8) media (Korsmeyer et al., 1983).

### Drug release kinetics

Drug release kinetics was examined by employing the following kinetic models (Korsmeyer et al., 1983; Peppas, 1985) with the help of Sigma Plot software as shown in (Table 3);

➢ Zero order model
➢ First order model
➢ Higuchi model
➢ Korsmeyer Peppas model
➢ Hixon and Crowell model

**TABLE 3:** Models to ascertain the kinetic of drug release.

Mathematical model	Equation	Diffusion exponent (n)	Mechanism of drug release
Zero order	𝑄𝑡 = 𝑄_0_ +𝐾_0_𝑡	0.45	Fickian diffusion
First order	ln𝑄_𝑡_ = ln𝑄_0_ +𝐾_1_𝑡	0.45 < n < 0.89	Anomalous (Non-Fickian) diffusion
Higuchi model	𝑄𝑡 = 𝐾_𝐻_𝑡^1/2^	0.89	Case II transport
Korsmeyer Peppas model	𝑄_0_^1/3^ − 𝑄_𝑡_^1/3^ = 𝐾_𝑠_𝑡	n > 0.89	Super Case II transport
Hixon and Crowell model	𝑄_𝑡_/𝑄_∞_ = 𝐾_𝑘_𝑡_𝑛_		

The value of *n* describes the mechanism of release of drug as given in Table 3.

### Accelerated stability studies

The developed formulation was placed in amber color vial and sealed with aluminum cap. The accelerated stability study was carried out for short period of time according to ICH guidelines to analyze the sample for drug content, density, pH, and gelling capacity that is done every month (Shastri et al., 2010b; Khorasani et al., 2021).

### FTIR spectroscopy

FTIR spectra of pure drug, HPMC, acrylic acid and glycerol were recorded to study the interaction between drug and excipients on FTIR spectrophotometer (IR Prestage 21, Shimadzu) in the range of 4,000–400 cm^−1^ using KBr mixing method with a resolution of 4 cm^−1^ for 20 scans (Chavda et al., 2013).

### Differential scanning calorimetry (DSC)

Differential scanning calorimeter (SDT Q-600) was used to characterize the thermal behavior of the drug and release retardant polymer by recording DSC thermograms in which nitrogen flow rate and linear heating rate was kept 40 ml/min and 10 C/min respectively and samples were heated between 30 and 300°C (Chavda et al., 2013).

### Scanning electron microscopy (SEM)

Scanning electron microscope (Quanta 250 Maker Fei) was used to study surface morphology of SPH by placing the transverse section of dried SPH samples on a double sided tap on aluminum stubs and a gold was coated on it by ion sputter (JEOL) (Bhalla, Nagpal).

### Analgesic activity of superporous hydrogels (SPH-17)

The analgesic activity of superporous hydrogel formulation SPH-17 was also analyzed by using a tail flick method. All the experimental methods involving animals were performed according to guidelines of UK Animals (Scientific Procedures) Act 1986 and approved by the Ethical Committee (UE/S&T/2020/75) of University of Education, Lahore-Pakistan. For this purpose, two six member groups of albino male and female mice having an average weight of 35 g were created. The group-I was given suspension of Mefenamic acid while group-II was given the selected formulation of hydrogels. Just 12 h before starting study, food was withdrawn while maintaining animals at room temperature. The calculated dose of drug was orally administered in both groups just 30 minutes before initiating the study. The animals were fixed on tail flick apparatus of analgesia meter for evaluating analgesic activity. The tail was inserted in the sensing groove above the photo-sensor. On the distal part of tail, the beam generated from radiant heat stimulus was focused and time taken by animal to withdraw the tail was calculated as the reaction time of the analgesic effect. Every trial was taking a time of 10 s as cut-off time to avoid tissue damage. The time of reaction for analgesic effect was measured after every hour for 10 h and any change in mice behaviour in both groups was detected (Ibrahim et al., 2010; Saleem et al., 2011).

## Results and discussion

### Porosity, viscosity, drug content and swelling ratio

The porosity viscosity and drug contents in all seventeen formulations were calculated and found to be in range of 36.70%–51.75%, 210 cps–548 cps, 61.96%–91.36% (Table 4). The porosity and viscosity of a formulation play a vital role in controlling the drug release from a formulation and the formulation SPH-17 was observed to exhibit maximum porosity (51.75%) and viscosity (548 cps). The parameter drug content represents the amount of drug entrapped in hydrogels through gas blowing method. The drug contents in SPH-17 formulated with 2.5% of acrylic acid was found to be 83% while formulation SPH-10 showed a little bit higher drug entrapment of 85% because of use of higher amount of acrylic acid (3%). Another critical parameter controlling the drug release from a formulation is swelling ratio which was calculated in water, Phosphate Buffer (pH 6.8) and in 0.1 N HCl (PH 1.2). The swelling ratio was observed to be in range of 2.31%–5.58%, 1.40%–2.60%, and 0.25%–1.15% in water, Phosphate Buffer and in 0.1 N HCl respectively. A good swelling behaviour of superporous hydrogels in all media of study clearly demonstrated the optimum capability of SPHs to control the availability of drug from formulation and the role of cross linker acrylic acid was obderved to be significant in inducing the porosity and swelling in SPHs.

**TABLE 4:** Measured values of porosity, viscosity, drug content, swelling ratio (water), swelling ratio [phosphate buffer (pH 6.8)] and swelling ratio [0.1 NHCL (pH 1.2)] of 17 superporous hydrogel formulations.

Formulations	Porosity^a^ (%)	Viscosity^a^ (cps)	Drug content (%)	Swelling ratio (Water)^a^	Swelling ratio (phosphate Buffer)^a^	Swelling ratio (0.1 NHCL)^a^
SPH 1	36.93	210	70.69	2.73	1.75	0.25
SPH 2	39.75	280	91.36	2.79	1.73	0.96
SPH 3	42.57	310	65.32	2.81	1.80	0.17
SPH 4	41.79	340	86.32	3.08	2.3	1.2
SPH 5	41.00	318	61.96	3.31	1.40	0.55
SPH 6	40.38	323	65.32	3.00	1.72	1
SPH 7	39.75	285	82.96	2.64	2.14	1.14
SPH 8	38.23	266	70.36	2.51	2.23	1
SPH 9	36.70	250	46.00	2.31	2.45	1.16
SPH 10	39.57	300	85.48	4.16	1.40	1.11
SPH 11	42.45	403	82.96	5.58	1.50	0.82
SPH 12	45.12	363	49.36	4.66	1.63	0.85
SPH 13	47.79	323	65.32	3.31	1.88	1.13
SPH 14	45.98	418	82.96	3.16	2.17	0.25
SPH 15	44.17	511	77.33	2.58	2.6	0.68
SPH 16	47.96	534	89.68	2.92	2.1	1.15
SPH 17	51.75	548	82.96	2.81	2.4	1.2

^a^ Average of three determinations.

### Optimization of experimental variables

For response surface methodology, a three-factor, five-level central composite rotatable design requires 17 experiments. All of the prepared formulations showed a good fit with quadratic model as observed by using software Design Expert. A positive value represents an issue that favors the optimization, while a negative value indicates an opposite relationship between the formulation variable and the studied response. The Eqs 6–11 depicted the quantitative influence of process variables; X_1_ (acrylic acid), X_2_ (HPMC) and X_3_ (glycerol) and their interactions on the responses Y_1_ (Porosity), Y_2_ (Viscosity), Y_3_ (Drug Content), Y_4_ [Swelling ratio (water)], Y_5_ [Swelling ratio (Phosphate Buffer (pH 6.8)], Y_6_ [Swelling ratio (0.1 N HCl (pH 1.2)].

$$Y_{1}\left(Porosity\right)=+33.07+0.35X_{1}+0.64X_{2}-0.35X_{3}-0.56X_{1}X_{2}-0.52X_{1}X_{3}-1.38X_{2}X_{3\;}-4.65X_{1}^{2}-2.66X_{2}^{2}-1.56X_{3}^{2}$$ (6)

$$Y_{2}\left(Viscosity\right)=+542.77+12.45X_{1}+0.20X_{2\;}+15.51X_{3}-8.00X_{1}X_{2}-14.25X_{1}X_{3\;}-31.25X_{2}X_{3\;}-102.51X_{1}^{2}-64.33X_{2}^{2}-68.75X_{3\;}^{2}$$ (7)

$$Y_{3}\left(Drug\;Content\right)=+98.74+7.24X_{1\;}-2.99X_{2\;}-0.25X_{3}-1.95X_{1}X_{2\;}-6.36X_{1}X_{3\;}-4.55X_{2}X_{3\;}-4.78X_{1}^{2}-4.63X_{2}^{2}-1.81X_{3}^{2}$$ (8)

$$Y_{4}\left(Swelling\;Ratio-Water\right)=+2.81+0.22X_{1}-0.17X_{2}-0.015X_{3}+0.049X_{1}X_{2}-0.096X_{1}X_{3}-0.19X_{2}X_{3}-0.064X_{1}^{2}+0.60X_{2}^{2}-0.064X_{3}^{2}$$ (9)

$$Y_{5}\left(Swelling\;Ratio-Buffer\right)=+2.48-0.064X_{1}+0.15X_{2}+0.029X_{3}+0.036X_{1}X_{2}+8.75X_{1}X_{3}+0.079X_{2}X_{3}-0.18X_{1}^{2}-0.31X_{2}^{2}-0.14X_{3}^{2}$$ (10)

$$Y_{6}\left(Swelling\;Ratio-0.1NHCL\right)=+1.10+0.14X_{1}+0.059X_{2}-0.027X_{3}-0.034X_{1}X_{2}-0.18X_{1}X_{3}+0.054X_{2}X_{3}-6.613X_{1}^{2}-0.11X_{2}^{2}-0.16X_{3}^{2}$$ (11)

The values of coefficients with the one factor tells about the intensity of effect of that particular factor on a response and the values of coefficients with multiple factors and second order terms explain the strength of interaction of factors under study and they have exhibited the quadratic quality of the this RSM phenomena.

The values of the coefficients in the equations for the independent variable X_1_ (acrylic acid) indicated positive effect upon the porosity, viscosity, drug content, swelling ratio in water, and 0.1 N HCl (pH 1.2) and negative impact upon the swelling ratio in phosphate buffer (pH 6.8). The values of the coefficients in the equations for the independent variable X_2_ (HPMC) indicated positive impact upon the porosity, viscosity, swelling ratio in Phosphate buffer (pH 6.8), and 0.1 N HCl (pH 1.2), and negative effect upon the drug content, and swelling ratio in water. Similarly, the values of the coefficients in the equations for the independent variable X_3_ (glycerol) indicated positive effect upon the viscosity, swelling ratio in phosphate buffer (pH 6.8), and negative impact upon the porosity, drug content, swelling ratio in water, and 0.1 N HCl (pH 1.2). The interaction terms (X_1_X_2_, X_1_X_3_ and X_2_X_3_) showed negative influence upon the porosity and viscosity, and positive influence upon the swelling ratio in phosphate buffer (pH 6.8) as represented by the values of the response coefficients in the above equations. The interaction term (X_1_X_2_) showed negative influence upon the drug content and swelling ratio in 0.1 N HCl (pH 1.2). The interaction term (X_1_X_3_) showed negative influence upon the drug content and swelling in 0.1 N HCl (pH 1.2) respectively while interaction term (X_2_X_3_) had showed positive impact upon the drug content and swelling in 0.1 N HCl (pH 1.2) respectively as represented by the values of the response coefficients in the equation. The interaction terms (X_1_X_3_ and X_2_X_3_) had showed negative influence upon the swelling ratio in water while interaction term (X_1_X_2_) had showed positive influence upon the swelling ratio in water as represented by the values of the response coefficients in the equation.

Regarding the involvement of quadratic terms (X_1_^2^, X_2_^2^, and X_3_^2^), it indicated that the increase in the concentration of the independent formulation variables caused an increase in porosity, viscosity and drug content of SPHs up to maximum but after that these parametrs were found to be decreased. Similarly, the involvement of quadratic terms (X_1_^2^, X_3_^2^) indicated that the increase in their concentration - caused an increase in swelling ratio in water up to maximum after that it - was decreased, while the quadratic term (X_2_^2^) indicates the decrease in the value of swelling ratio in water up to minimum after that it was increased. The increase in concentration of quadratic terms (X_2_^2^) caused an increase in swelling ratio of SPHs in phosphate buffer (pH 6.8) up to maximum after that it was decreased and for the quadratic term (X_1_^2^), the increase in its concentration caused an increase in swelling ratio of SPHs in 0.1 N HCl (pH 1.2) up to maximum after that it was decreased (Pabari and Ramtoola, 2012).

**FIGURE 1:**
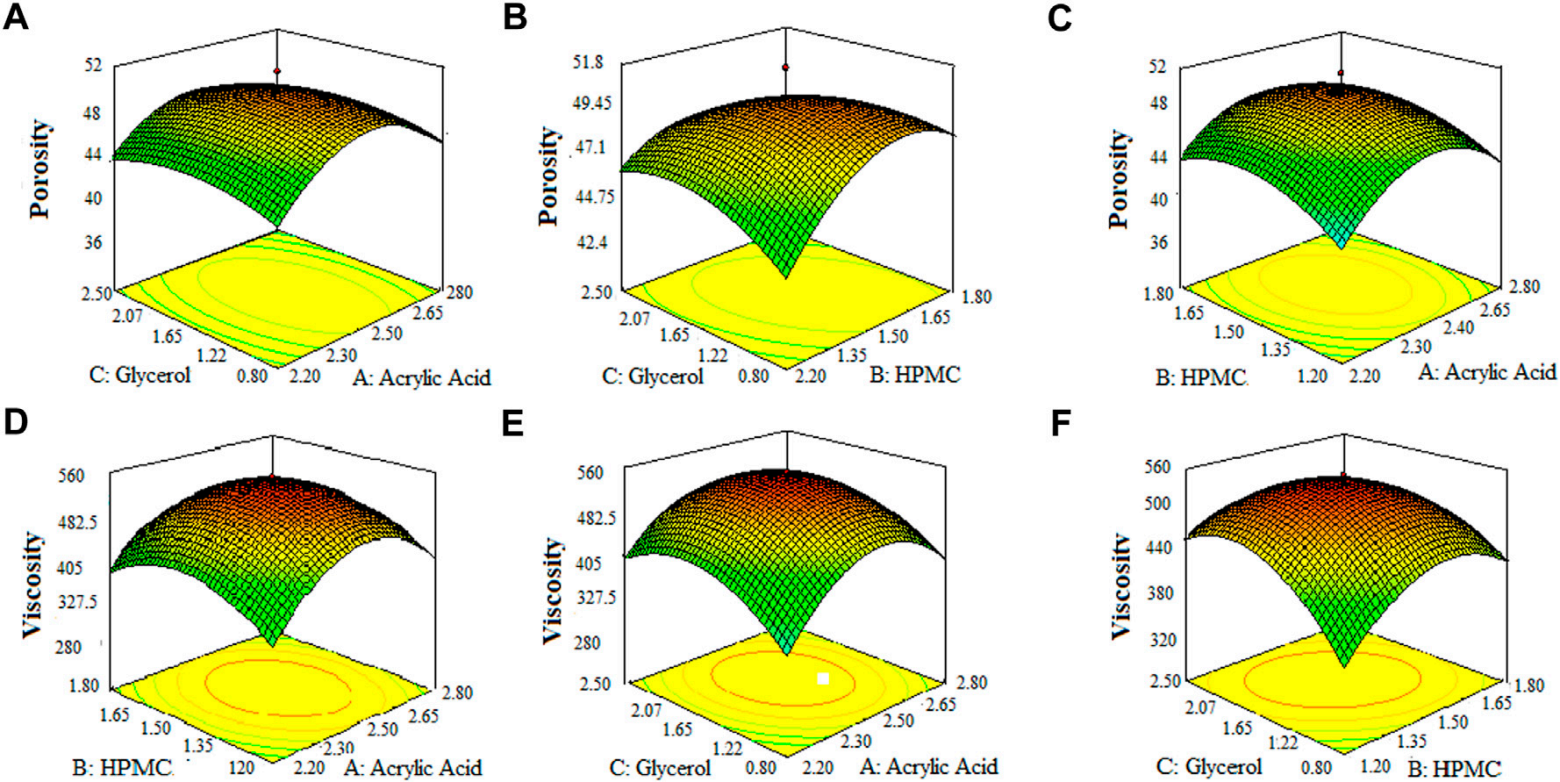
3D surface plots indicating the effect of various concentration of hpmc-k100, glycerol and acrylic acid on porosity **(A–C)** and viscosity of SPHs **(D–F)**.

**FIGURE 2:**
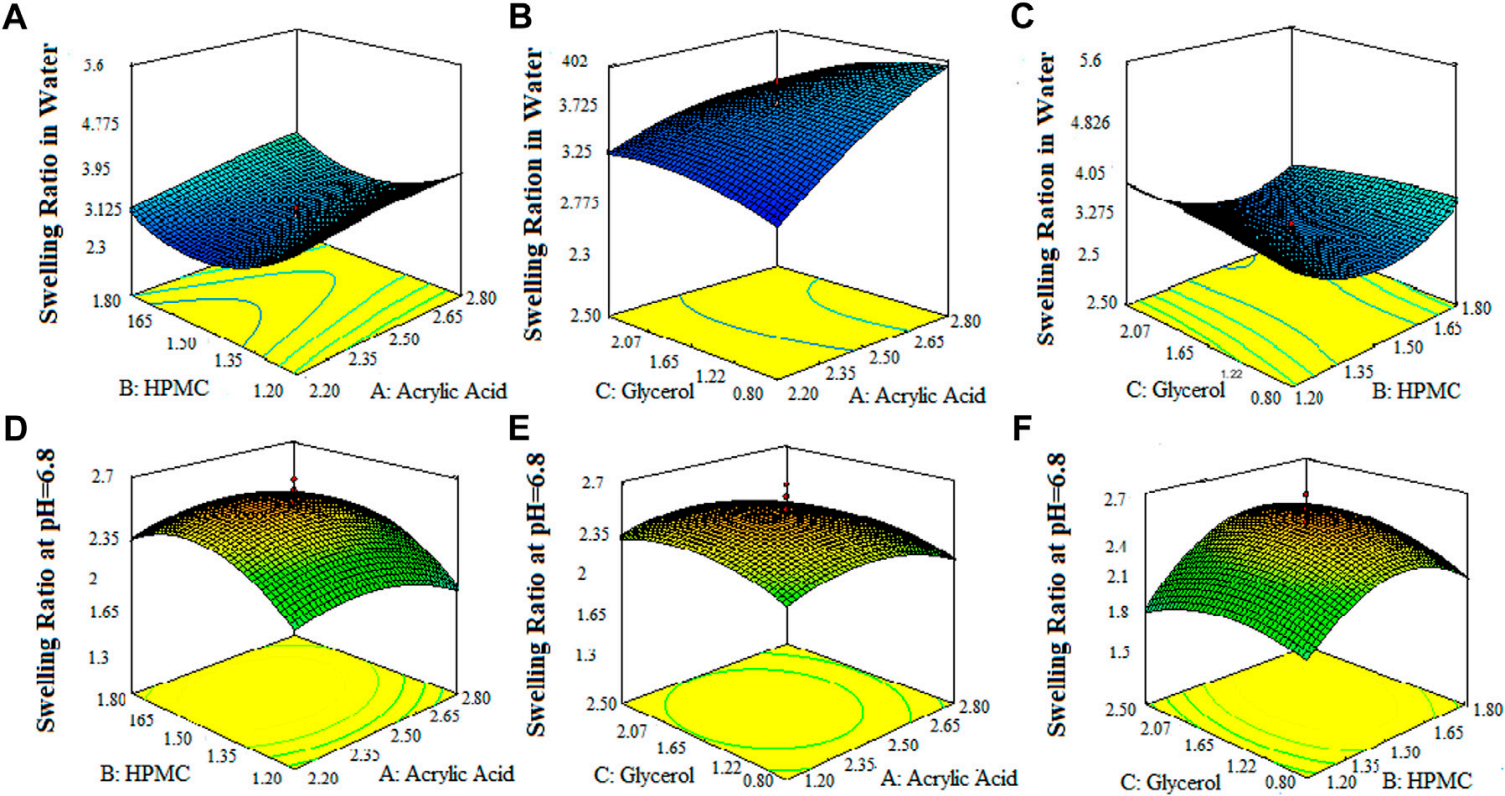
3D surface plots indicating the effect of various concentration of HPMC-k100, glycerol and acrylic acid on drug content swelling ratio of SPHs in water **(A–C)** and swelling ratio of SPHs in buffer pH = 6.8 **(D–F)**.

**FIGURE 3:**
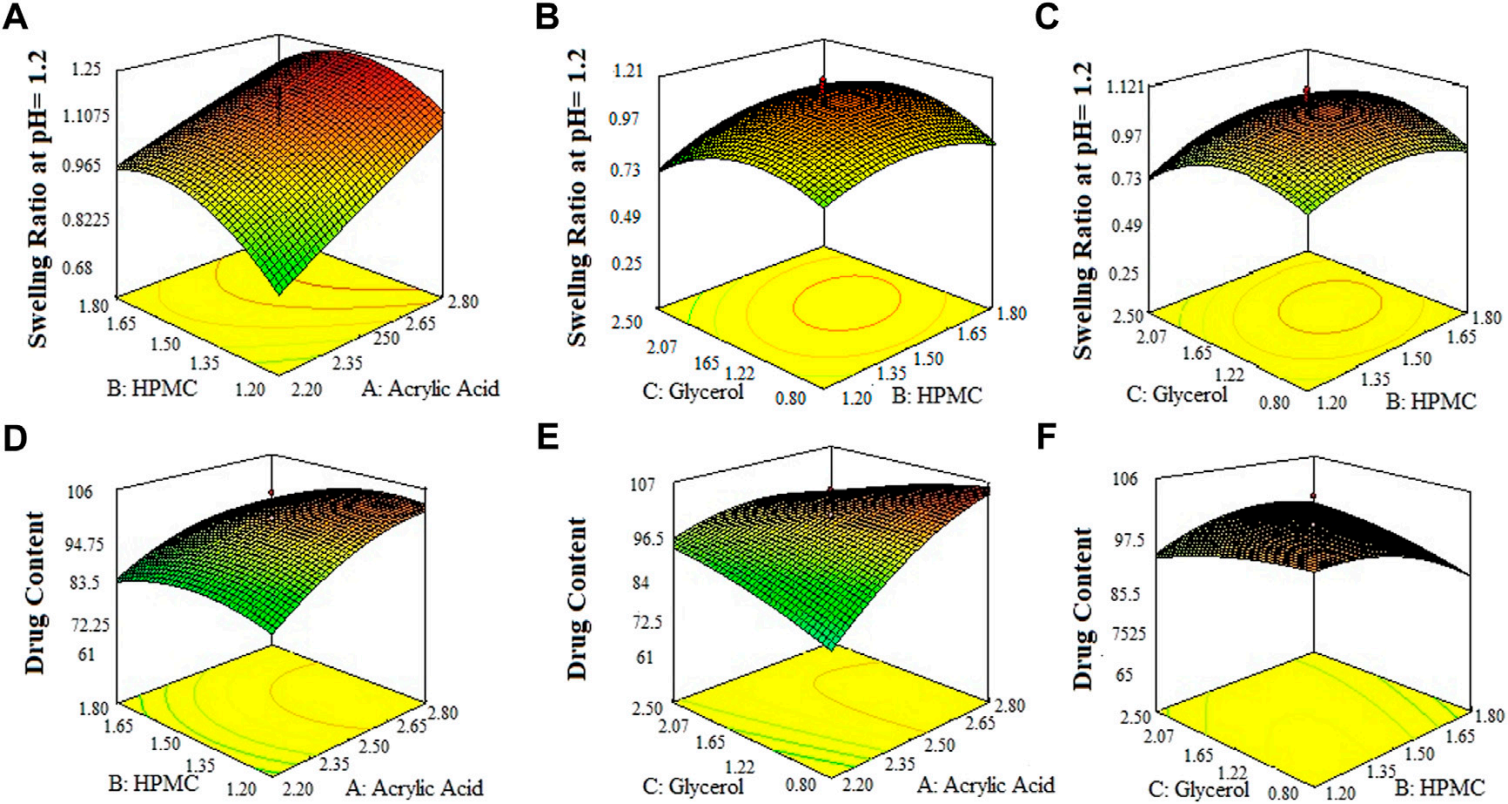
3D surface plots indicating the effect of various concentration of HPMC-K100, glycerol and acrylic acid on swelling ratio of SPHs in 0.1 N HCl pH = 12 **(A–C)** and drug content in SPHs **(D–F)**.

The 3-D surface plots indicated that increase in concentration of X_1_ (acrylic acid) from 2.2% to 2.5% caused an increase in porosity (42%–50%), and viscosity (365.07–508.155 cps), while the increase in concentration of X_1_ (acrylic acid) from 2.5 to 2.8% cause an increase in drug content (68.8%–89.6%), swelling ratio in water (2.7%–2.92%), and in 0.1 N HCl (0.82%–1.2%). It caused a decrease in swelling ratio in phosphate buffer from 2.6% to 1.8%) of the SPH formulations. They also determined that the increase in concentration of X_2_ (HPMC) from 1.2% to 1.5% brought an increase in viscosity (386.585 cps–449.205 cps), porosity (45%–52%), and swelling ratio in phosphate buffer (1.6%–1.9%) and decrease in drug content (87.3%–70.2%) and in swelling ratio in water (2.85%–2.69%) and showed no significant effect upon the swelling ratio in 0.1 N HCl (0.96%–1.1%) of the formulations. They also showed that the increase in concentration of X_3_ (glycerol) from 0.8% to 1.65% results in increase in viscosity from 392.886 cps to 483.333 cps), and swelling ratio in 0.1 N HCl (pH 1.2) and decrease in drug content from 85.5% to 67.9% while it showed no significant effect upon the swelling ratio in phosphate buffer (pH 6.8) (2.1%–2.3%) and swelling ratio in water (2.7%–2.89%), and porosity (43.6%–45.8%) of the prepared formulations as shown in the Figures 1–3. The values of the coefficients of the variables and 3D plots had determined that the X_2_ (HPMC) has greater influence upon the porosity, and swelling ratio in phosphate buffer (pH 6.8) of the formulations. The X_1_ (acrylic acid) has greater influence upon the drug content, viscosity, and swelling ratio in water while X_2_ (HPMC) has shown insignificant impact upon the drug content, and swelling ratio in water of the formulations. The X_1_ (acrylic acid) and X_2_ (HPMC) has significant effect upon the swelling ratio in 0.1 N HCl (pH 1.2) and viscosity but the influence of X_1_ (acrylic acid) is more persuasive than X_2_ (HPMC) upon the swelling ratio in 0.1 N HCl (pH 1.2) and viscosity of the formulations (Gannu et al., 2009).

### Density, pH and void fraction

The superporous hydrogels (SPHs) were detected to be low dense as they expressed density lower than 0.75 g/cc. The density of the formulation may be affected by the concentration of the polymer-HPMC as the density of the formulation increases with an increase in the concentration of the polymer in the formulation (Bhalla, Nagpal). The formulations SPH-1 to SPH-3 have lower values of density and the formulation SPH-4 to SPH-17 has the higher values of density as shown in the (Table 5). The increasing concentration of HPMC from 1.2 to 1.80%. Acrylic acid from 2.20 to 2.80% and glycerol from 0.80 to 2.50% contributed a lot to bring an increase in density - of seventeen SPH formulations. The results were also evaluated statistically by applying ANOVA with the help of Minitab software that provide the *p*-value for density (*p* = 0.569) that was greater than 0.05 showing the insignificancy of the results. The statistical evaluation tells us that the increasing concentration of the polymers contributed towards the higher density values of the SPH formulations (SPH 1 to SPH 17).

**TABLE 5:** Results of density, pH, Void fraction and *in vitro* gelling capacity of superporous hydrogel formulations in 0.1 NHCL (pH 1.2) and phosphate buffer (pH 6.8).

Formulations	Void fraction^a^	Density^a^	pH	*In Vitro* gelling capacity	
				0.1 N HCl (pH 1.2)	Phosphate buffer (pH 6.8)
SPH 1	4.5	0.55	5.3	+++	+
SPH 2	2.8	0.57	4.0	+++	+
SPH 3	1.6	0.59	5.1	+++	+
SPH 4	8.4	0.63	6.0	+++	++
SPH 5	2.2	0.67	5.5	+++	++
SPH 6	1.8	0.67	5.5	+++	++
SPH 7	1.0	0.68	5.6	++	++
SPH 8	2.6	0.66	5.8	+++	++
SPH 9	2.4	0.63	6.5	+++	++
SPH 10	2.2	0.62	5.7	+++	++
SPH 11	2.0	0.60	4.4	+++	++
SPH 12	2.4	0.66	5.6	+++	++
SPH 13	2.6	0.73	5.6	++	++
SPH 14	2.7	0.70	5.1	+++	+
SPH 15	3.9	0.66	5.9	++	+
SPH 16	4.4	0.67	4.3	++	+
SPH 17	5.0	0.68	6.1	++	+

^a^ Average of three determinations.

+, Gelation occurred after few minutes and gel dissolved rapidly; **++**, gelation went on immediately and stays for up to 8 h; **+++**, gelation took place quickly and persists for more than 10 h.

The values observed for void fraction were found to be non-significant for cross linked formulations. The higher value of the void fraction contributed towards the decreased swelling of the SPH particles that ultimately causes the decrease uptake of water into the SPH structures. It resulted in the decrease swelling ratio of SPH particles (Gupta and Shivakumar, 2012). The formulations SPH-3, SPH-6, and SPH-7 exhibited the lowest values of void fraction as containing the highest concentration of HPMC (1.80%), acrylic acid (2.8%) and glycerol (2.50%) as shown in the Table 5. The formulations SPH-1, SPH-2, SPH-4, SPH-5, and SPH-8 to SPH-17 had presented the highest values of void fraction as containing the lowest concentration of HPMC (1.2%–1.5%), acrylic acid (2.2%–2.5%) and glycerol (0.8%–1.65%) as tabulated in the Table 5. The results were also evaluated statistically by applying ANOVA with the help of Minitab software that provide the *p*-value for void fraction (*p* = 0.202) that were greater than 0.05 showing the insignificancy of the results. The statistical evaluation tells us that the decreased concentration of the polymers and cross linker contributed towards the increase in the void fraction of the SPH formulations. The pH of the superporous hydrogels was determined to be in the range of 4–6.5 that was close to the gastric pH, providing the gastric retention application of the formulations as tabulate in the Table 5 (Khorasani et al., 2021).

### *In Vitro* gelling capacity study

The *in vitro* gelling capacity of seventeen formulations was performed in 0.1 N HCl (pH 1.2) and phosphate buffer (pH 6.8). All formulations undergone immediate gelling as they come into the contact with 0.1 N HCl (pH 1.2) and phosphate buffer (pH 6.8) (Khorasani et al., 2021). The *in vitro* gelling capacity was observed to be maximum for all SPH formulations in 0.1 N HCl (pH 1.2) as presented in the Table 5. The *in vitro* gelling capacity of SPH 4 to SPH 13 was found to be maximum in phosphate buffer (pH 6.8) while the *in vitro* gelling capacity of SPH 1 to SPH 3 and SPH 14 to SPH 17 was found to be minimum in phosphate buffer (pH 6.8) as shown in the Table 5.

### *In Vitro* drug release study

**FIGURE 4:**
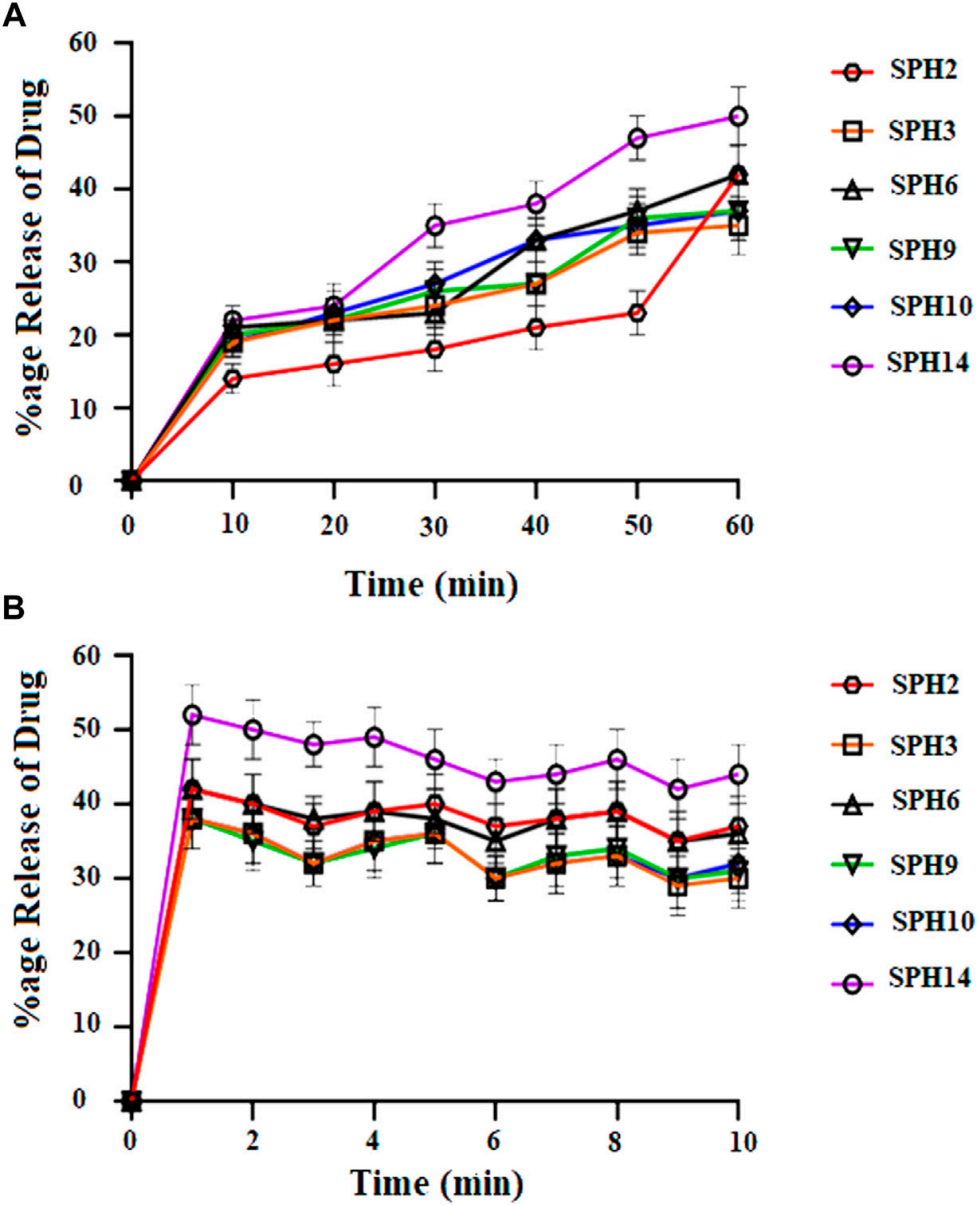
Cumulative Percent release of Mefenamic acid from the Superporous Hydrogels formulations (SPH 2, SPH 3, SPH 6, SPH 9, SPH 10, and SPH 14) in O.1 NHCL **(A)** and in phosphate buffer-pH 6.8 **(B)** *n* = 3.

**FIGURE 5:**
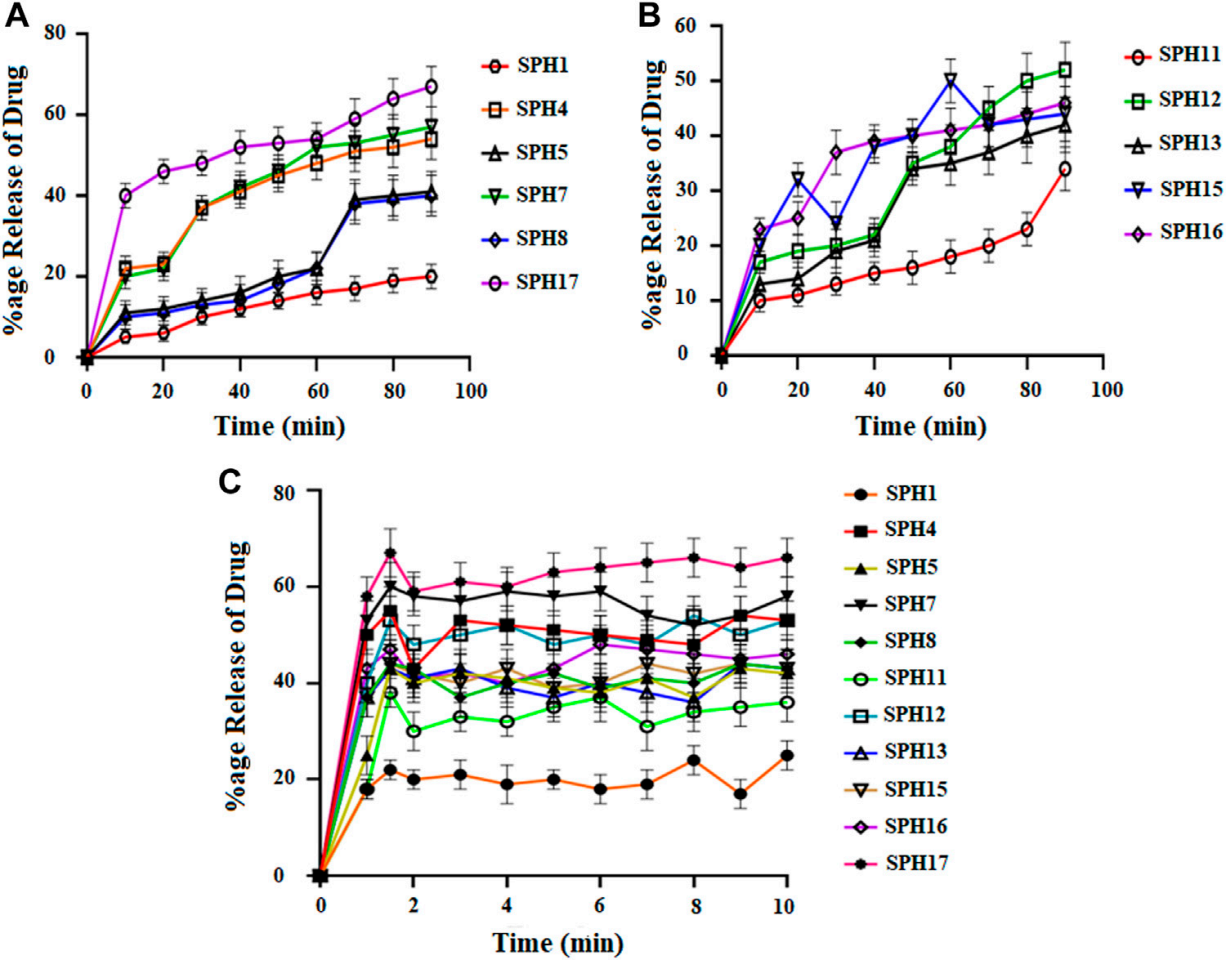
Cumulative Percent release of Mefenamic acid from the Superporous Hydrogels formulations (SPH 1, SPH 4, SPH 5, SPH 7, SPH 8, and SPH 17) **(A)** (SPH 11, SPH 12, SPH 13, SPH 15, SPH 16) **(B)**, in O.1 NHCL and (SPH 1, SPH 4, SPH 5, SPH 7, SPH 8, SPH 11–13, and SPH 15–17) **(C)** in phosphate-buffer-pH 6.8 (*n* = 3).

**FIGURE 6:**
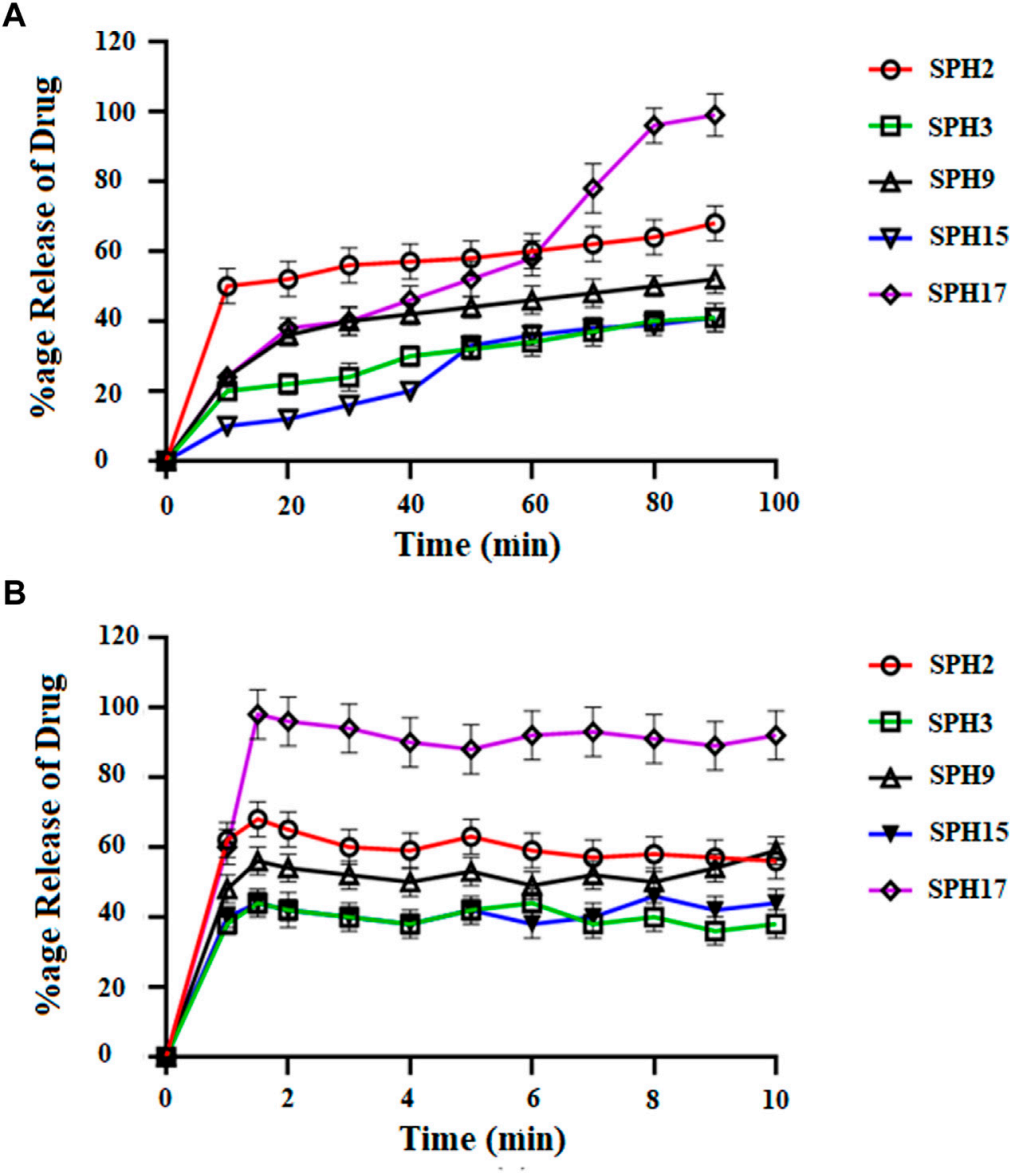
Cumulative Percent release of drug from Superporous Hydrogel formulation (SPH 1, SPH 4–8, SPH 10–14, and SPH 16) in 0.1 NHCL **(A)** and in phosphate buffer-pH 6.8 **(B)** *n* = 3.

A non-significant drug release was observed from the seventeen superporous hydrogel formulations (SPH 1 to SPH 17). There was an initial rapid release of drug from the formulations (SPH 1, SPH 4-SPH 8, and SPH 10-SPH 16) for 60 min in 0.1 N HCl. There was an initial rapid release of drug from the formulations (SPH 2, SPH 3, SPH 6, SPH 9, SPH 10, and SPH 14) for 60 min in phosphate buffer (pH 6.8) as presented in the Figures 4, 5. There was also an initial rapid release of drug from the formulations (SPH 2, SPH 3, SPH 9, SPH 15, and SPH 17) for 90 min in the 0.1 N HCl as depicted in the Figure 6. There was an initial rapid release of drug from the formulations (SPH 1, SPH 4, SPH 5, SPH 7, SPH 8, and SPH 11-SPH 13, and SPH 15-SPH 17) for 90 min in the phosphate buffer (pH 6.8) as shown in the (Figures 4, 5). Then, the drug release occurred slowly for the further 10 h in both types of media as shown in the (Figures 4–6). The initial rapid release of drug form the SPH formulations in both media [0.1 N HCl and phosphate buffer (pH 6.8)] can be attributed to the presence of the drug to the exterior outer surface of the network structure of SPHs and then the drug release was occurred slowly by diffusion and relaxation of polymer present as incorporated within the hydrogel structure (Bhalla, Nagpal).

The decrease in the release of the drug from the SPH formulations (SPH 1, SPH 3-SPH 8, SPH 10-SPH 12, SPH 14, and SPH 15) in the 0.1 N HCl may be attributed to the increase in the swelling ratio of these SPH formulations that hinder the diffusion of the drug from the swelled structure. The decrease in the release of the drug from the SPH formulations (SPH 1-SPH 3, SPH 5, SPH 6, SPH 8-SPH 11, SPH 13, SPH 15 and SPH 16) in the phosphate buffer (pH 6.8) may also be attributed to the increase in the swelling ratio of these SPH formulations that hinder the diffusion of the drug from the swelledhydrogels.

The increase in the concentration of the HPMC, acrylic acid and glycerol from the 1.5%–1.8%, from the 2.5%–2.8% and from the 0.80%–1.65% respectively contribute to the higher swelling of these SPH formulations in the both media. The SPH formulations likeSPH 2, SPH 9, SPH 13, SPH 16, and SPH 17 showed the significant drug release of 67.5, 58.5, 51.3, 56.7, and 99.8% respectively in the 0.1 N HCl while the SPH formulations like SPH 4, SPH 7, SPH 12, SPH 14 and SPH 17 showed the significant release of drug of 55.8, 60.3, 53.1, 51.3, and 66.6% respectively in the phosphate buffer (pH 6.8). The significant release of the drug from the SPH formulations in the 0.1 N HCl as compared to the phosphate buffer may be attributed to the better mechanical strength of the SPH in the HCl medium that prevents the collapsing of the interconnected channels and allow the better diffusion of the drug from the formulations (Khorasani et al., 2021). The increase in the concentration of HPMC from 1.2 to 1.5%, Acrylic acid from the 2.2–2.5% and glycerol 1.65–2.50% results in the formation of more entangled type of polymeric network that causes the sustained release of drug from the SPH formulations. The water cannot penetrate into the polymeric network results in the reduction of dissolution and erosion rates. The high polymeric network concentration also increased the density of the formulations and hindered the release of the drug from the formulation (Bhalla, Nagpal). The SPH 17 formulation was the optimized formulation that achieved the significant release of drug in the both 0.1 N HCl (99.8%) and phosphate buffer (pH 6.8) (66.6%) media as SPH-17 containing the optimized concentrations of polymers (HPMC, 1.5%), monomer (acrylic acid, 2.50%) and cross linker (glycerol, 1.65%).

The *in vitro* drug release profiles of seventeen SPH formulations (SPH-1 to SPH-17) was subjected to various drug release kinetic models such as zero order, korsemeyer peppas, first order, higuchi, and hixson and crowell model as shown in (Tables 6,7). The seventeen SPH formulations (SPH-1–SPH-17) in both media including 0.1N HCl and phosphate buffer (pH 6.8) followed the Korsemeyer-Peppas kinetic model as indicated by the higher *R* ^2^ value achieved by the SPH formulations for the Korsemeyer-Peppas kinetic model. The value of k increases with the increase in the concentration of the polymer. All SPH formulations (SPH1-SPH17) showed increase in the value of k as containing the increasing concentration of the HPMC and acrylic acid from 1.2 to 1.50% and 2.2%–2.80% respectively as shown in the Tables 6, 7 (Khorasani et al., 2021). Both type of drug release mechanisms such as fickian and non-fickian were followed by the seventeen SPH formulations (SPH1-SPH17) in both type of release media as indicated by the value of *n* (Tables 6,7). The non-fickian release of drug reveals the coupling of the diffusion and polymer relaxation mechanism indicating the different release mechanisms of the drug from the formulations (Saleem et al., 2011).

**TABLE 6:** Model dependent approach of superporous hydrogel formulations in phosphate buffer (pH 6.8).

Formulations	Zero order		Korsemeyer peppas			Hixon and crowell		Higuchi		First order	
	*R* ^2^	k_0_	*R* ^2^	k_k_	n	*R* ^2^	k_s_	*R* ^2^	k_H_	*R* ^2^	k_1_
SPH 1	0.9670	1.70	0.9914	0.91	0.72	0.9670	0.001	0.9566	7.27	0.9722	0.003
SPH 2	0.7540	3.7	0.8223	2.50	0.63	0.7643	0.002	0.9088	6.12	0.7689	0.007
SPH 3	0.4987	5.1	0.9499	6.30	0.42	0.4876	0.002	0.9382	5.80	0.5721	0.008
SPH 4	0.8649	6.6	0.9853	7.90	0.44	0.7555	0.003	0.9792	6.24	0.8324	0.011
SPH 5	0.9634	0.94	0.9683	0.76	0.88	0.9592	0.001	0.8932	7.97	0.9563	0.006
SPH 6	0.7363	6.3	0.9422	5.6	0.48	0.7270	0.003	0.9419	8.33	0.7704	0.010
SPH 7	0.9190	6.9	0.9834	5.8	0.52	0.9080	0.003	0.9924	6.61	0.9453	0.012
SPH 8	0.9771	1.57	0.9857	0.9	0.87	0.9769	0.002	0.9592	6.30	0.9757	0.006
SPH 9	0.3890	0.05	0.7455	11.8	0.26	0.0073	0.002	0.9809	8.01	0.0094	0.009
SPH 10	0.4581	6.3	0.9607	7.4	0.40	0.3473	0.003	0.9380	5.18	0.4981	0.009
SPH 11	0.7570	1.86	0.9029	0.88	0.78	0.888	0.001	0.9606	6.87	0.8884	0.004
SPH 12	0.9654	3.34	0.9721	2.10	0.71	0.9573	0.002	0.9396	5.13	0.9614	0.008
SPH 13	0.9444	3.67	0.9747	2.58	0.64	0.9381	0.002	0.9577	6.57	0.9519	0.007
SPH 14	0.9435	4.65	0.9828	5.73	0.53	0.8712	0.004	0.9811	6.56	0.9053	0.013
SPH 15	0.3976	8.77	0.8763	9.97	0.35	0.0038	0.002	0.9174	5.44	0.2894	0.009
SPH 16	0.8257	1.02	0.9720	11.7	0.31	0.0270	0.002	0.9469	5.56	0.0019	0.009
SPH 17	0.9789	0.09	0.9864	23.0	0.22	0.0421	0.004	0.9511	5.72	0.0230	0.016

**TABLE 7:** Model dependent approach of superporous hydrogel formulations in 0.1NHCL (pH 1.2).

Formulations	Zero order		Korsemeyer peppas			Hixon and crowell		Higuchi		First order	
	*R* ^2^	k_0_	*R* ^2^	k_k_	n	*R* ^2^	k_s_	*R* ^2^	k_H_	*R* ^2^	k_1_
SPH 1	0.5712	1.01	0.9329	16.5	0.27	0.0321	0.002	0.6235	4.5	0.642	0.008
SPH 2	0.7643	4.5	0.9591	36.3	0.12	0.0271	0.005	0.0000	8.2	0.8894	0.015
SPH 3	0.5785	2.3	0.9753	5.21	0.48	0.7920	0.002	0.8804	5.9	0.8346	0.008
SPH 4	0.8855	6.7	0.8936	2.48	0.67	0.8543	0.002	0.8712	4.7	0.8587	0.008
SPH 5	0.9696	4.7	0.9774	8.39	0.42	0.5740	0.123	0.9564	6.2	0.6822	0.012
SPH 6	0.7465	7.8	0.9258	12.3	0.29	0.0761	0.003	0.7222	5.7	0.0340	0.011
SPH 7	0.9289	7.7	0.9813	35.8	0.06	0.0270	0.004	0.8930	7.4	0.0129	0.016
SPH 8	0.9414	3.68	0.9967	0.38	0.79	0.9303	0.002	0.8340	3.5	0.9251	0.006
SPH 9	0.4567	4.32	0.9867	12.0	0.34	0.0076	0.003	0.9147	6.4	0.4258	0.012
SPH 10	0.4769	7.6	0.9859	0.42	0.85	0.9854	0.001	0.8958	4.3	0.9849	0.004
SPH 11	0.7770	6.93	0.9371	10.4	0.33	0.0321	0.003	0.8510	5.7	0.0250	0.011
SPH 12	0.9225	7.25	0.9786	15.5	0.25	0.0120	0.013	0.5096	6.3	0.0371	0.128
SPH 13	0.9455	6.79	0.9522	21.8	0.19	0.0110	0.004	0.0000	7.3	0.0053	0.158
SPH 14	0.9527	4.44	0.9623	0.07	0.82	0.9293	0.001	0.7932	2.3	0.9248	0.004
SPH 15	0.5742	4.43	0.9693	1.67	0.75	0.9647	0.002	0.9248	4.7	0.9694	0.007
SPH 16	0.8569	5.87	0.9886	8.81	0.45	0.7604	0.004	0.9850	7.4	0.8360	0.016
SPH 17	0.9736	5.6	0.9876	3.3	0.74	0.9464	0.005	0.9369	9.1	0.9309	0.021

### Accelerated stability study

The three selected optimized formulations like SPH14, SPH16, and SPH17 were subjected to accelerated stability study by storing them at temperature and humidity of 40 ± 2°C and 75 ± 5% respectively. Afterwards, the optimized formulations were tested for various evaluation parameters like density, pH, drug content, and gelling capacity. The three studied formulations did not exhibit any significant change for all these evaluation parameters.

### Statistical analysis

The data received for different responses was analyzed by the one way ANOVA using Design Expert Software (version 11.0). The values obtained are considered significant and non-significant based on the *p*-value either less than or greater than 0.05. It was concluded that results of porosity and viscosity are significant suggesting the strong influence of formulation variables on viscosity and porosity while the results of drug content and swelling ratio in water, phosphate buffer (pH 6.8) and 0.1 N HCl (pH 1.2) were found to be non-significant. The *R* ^2^ values for all of the studied responses were also calculated by using Design Expert Software (version 11.0) and were shown in the Table 8 suggesting the goodness of the fit of the quadratic model for all the responses. The results obtained for the porosity are statistically significant as indicated by the *p*-value (*p* = 0.0038). Such values of porosity allow the proper flow of water through the capillaries formed into the SPH formulations. The values of the porosity also affect the swelling ratio of the SPH formulations to such an extent that maintain the mechanical strength of the SPH formulations and help in providing the sustained action of the drug from the SPH formulations. The results obtained for the viscosity are statistically significant as indicated by the *p*-value (*p* = 0.033). The HPMC and glycerol play a significant part in enhancing the viscosity of the SPH formulations. The heating applied during preparation of formulations and drying process significantly affect the viscosity of the HPMC that results in lowering the values of the viscosity of the SPH formulations. The results obtained for the drug content are statistically insignificant as indicated by the *p*-value (*p* = 0.186). The porosity of the SPH formulations affected the drug content of the SPH formulations. The higher values of the porosity result in lesser drug loading into the SPH formulations. Similarly, the results obtained for the swelling ratio in water, phosphate buffer (pH 6.8) and 0.1 N HCl (pH 1.2) were statistically insignificant as indicated by the *p*-values (*p* = 0.233, 0.099, and 0.425 respectively). The SPH formulations did not swell to a significant extent due to the lower values of the porosity. Such swelling helps in the lesser diffusion of the water through the SPH formulations and maintains the mechanical strength of the SPH formulations. The swelling ratio is more insignificant into the 0.1 N HCl (pH 1.2), then into the phosphate buffer (pH 6.8), and then into the water. This hierarchy of swelling ratio allowed the better maintenance of the mechanical strength of the SPH formulations in the respective medium that allow the better diffusion of the drug from the SPH formulations and sustained the drug action for a prolong period of time.

**TABLE 8:** Summary of Results of Regression Analysis for Porosity, Viscosity, Drug content, Swelling Ratio (water), Swelling ratio [phosphate buffer (pH 6.8)] and Swelling Ratio [0.1 NHCL (pH 1.2) for fitting to Quadratic Model.

	Porosity	Viscosity	Drug content	Swelling ratio (water)	Swelling ratio [phosphate buffer (pH 6.8)]	Swelling ratio [0.1 NHCL (pH 1.2)]
*R* ^2^	0.8528	0.9641	0.6187	0.5919	0.6796	0.5029
ANOVA (*p*-value)	0.0038	0.033	0.186	0.233	0.099	0.425

### FTIR study

**FIGURE 7:**
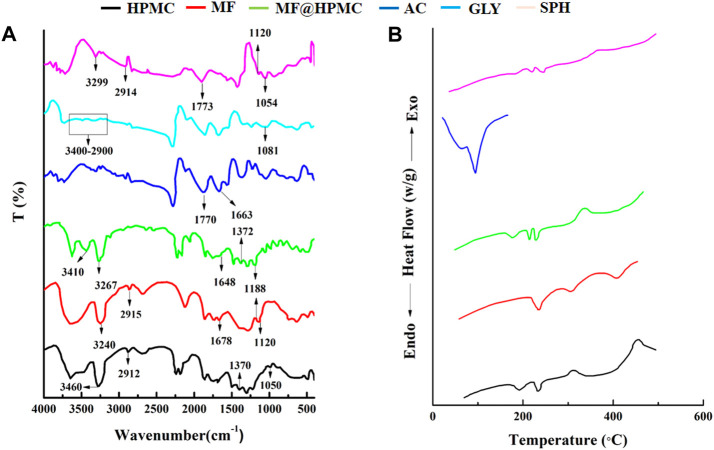
FTIR spectra of MF, HPMC, MF@HPMC, AC, GLY, and SPH formulations **(A)** and DSC thermograms of MF, HPMC, MF@HPMC, AC, GLY, and SPH formulations **(B)**.

The FTIR spectra of mefenamic acid (MF), acrylic acid (AC), HPMC, glycerol (GLY), superporous hydrogel (SHPs) were recorded and compared to ascertain the mode of interaction between various components of formulation as presented in Figure 7A. The FTIR spectrum of HPMC exhibited characteristics peaks at 3,422 cm^−1^ related O-H stretching frequency and at 1,370 cm^−1^ due to bending vibration of–OH. Other peaks found at 2,929 cm^−1^ and at 1,055 cm^−1^ indicated the presence of C-H and O-C bonds respectively (Iqbal et al., 2017; Iffat et al., 2020). The FTIR spectrum of MF presented characteristics peaks at 3,240 cm^−1^, 1,120/1,188 cm^−1^, 1,678 cm^−1^ relating to the presence of NH-H, C-N bond, and the COOH group respectively (Mudalip et al., 2013). The IR spectrum of combined from of MF and HPMC exhibited no new peak confirming their presence without any chemical interaction. The FTIR spectrum of glycerol depicted characteristics peaks at 2,900–3,400 cm^−1^ indicating the presence of H bonding and at 1,081 cm^−1^ indicated the presence of C-O functionality. The FTIR spectrum of acrylic acid had presented characteristics peaks at 1770 cm^−1^, and 1,663 cm^−1^ relating to the presence of α-β unsaturated groups, and the carboxylic group (COOH) respectively (Srivastava and Kumar, 2013). The presence of corresponding functional groups is same for the respective materials for the individual spectrum of these materials and for the spectrum of respective materials into the formulation SPHs that confirmed the presence of acrylic acid, HPMC, mefenamic acid, and glycerol in the hydrogel.

### DSC study

DSC thermograms of polymer (HPMC), drug (mefenamic cid), acrylic acid, physical mixture of polymer and drug and SPH formulation are presented in the Figure 7B. Pure HPMC and mefenamic acid exhibited peaks at 230°C and 232°C corresponding to their melting points, respectively. Likewise DSC of acrylic acid exhibited a sharp endothermic peak at 120°C at its melting point. (Malik et al., 2017). The same peaks also appeared at the corresponding melting points in the mixture of polymer and drug (MF@HPMC) as well as in the formulation (SPH) indicating no interaction between the polymer and the drug. A slight shifting and mild diffusion of the corresponding melting peaks of the polymer and drug in their physical mixture as well as in SPH formulation towards the higher temperature indicated that the polymer and drug had retained their pure crystalline form and processing conditions have not harmfully impacted the drug in final SPH (Li and Castillo, 2020; Anwar et al., 2021; Zahra et al., 2021).

### Scanning electron microscopy (SEM)

**FIGURE 8:**
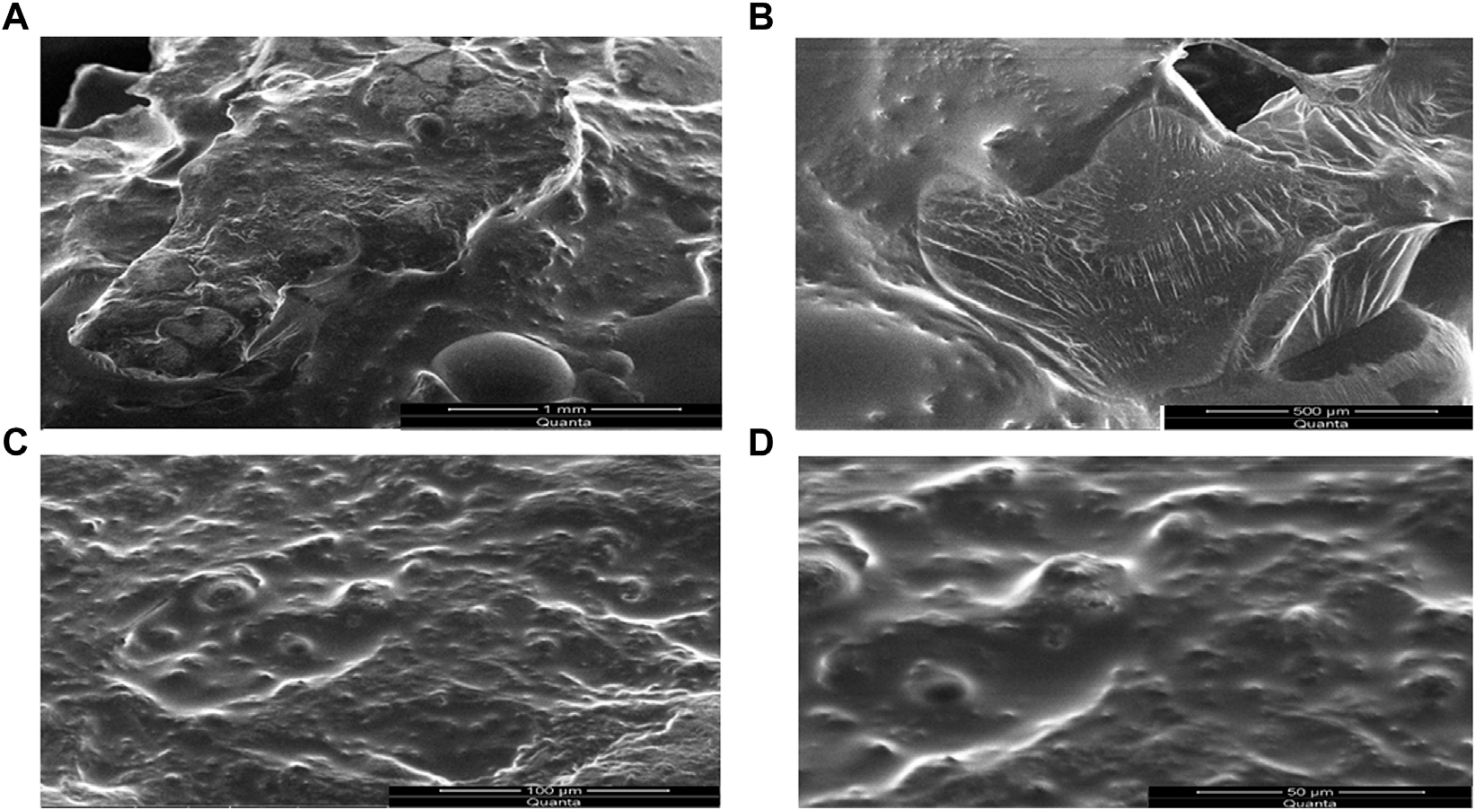
SEM micrographs of drug loaded SPHs formulated from Acrylic Acid/HPMC K100M and Glycerol.

The SEM micro graphs depicted the presence of pores on the surface of SPHs as presented in Figures 8A–D. The compression decreased the pores to some extent but does not affect the capillary structure of the SPHs. The SEM micrographs also showed the cluster type aggregates of drug and holes that are embedded in the smooth matrix structure of SPHs (8A). Drying of superporous hydrogels affected the collapsing and aggregation of the porous network of SPHs. The fast swelling of the hydrogels is due to the regular connectivity of the pores present in the structure (Figure 8B) which allowed the water and other fluids to pass through the structure by convection. The presence of the fibrous network interconnected the pores with each other but does not affected the porosity of the SPHs particles.

### Analgesic activity of superporous hydrogels (SPH-17)

The time taken by mice to withdraw tail was considered as the end point while keeping 10 s as the cut off time for preventing any tail injury. The group-I receiving suspension of drug induced the analgesic effect (4.2 ± 0.17 s) in 2 h with a time to achieve maximum analgesic effect in 4 h (7.1 ± 0.13 s). The analgesic effect in group-I remain maintained for 6 h. The poor, slow and delayed analgesic effect in group-I may be associated with poor absorption and poor bioavailability of mefenamic acid administered in suspension form. The analgesic effect of orally administered superporous hydrogels in group-II induced a significant effect in 1 h (4.9 ± 0.18 s) with an achievement of maximum pain relieving impact in 5 h (8.3 ± 0.12 s). The superporous hydrogels had maintained the analgesic effect for about 10 h. The study clearly depicted that hydrogels had not only release the drug for a prolonged time but also suggested that hydrogels had also contributed in the better absorption of drug from gastric mucosa (Korsmeyer et al., 1983; Desu et al., 2020). It was clearly manifested in the form of not only the rapid onset of action of drug but also in the form of prolonged sustained analgesic effect. The obtained outcomes suggested that analgesic effect of hydrogels was significantly (*p* < 0.05) higher than that of simple suspension of drug and total duration of analgesic effect for hydrogels was also doubled as compared to the duration of analgesic effect produced by the drug suspension.

## Conclusion

Super porous hydrogel system for the sustained delivery of mefenamic acid was prepared using various concentrations of polymers and cross linker. Central composite design was applied for optimizing the preparation parameters to obtain the prolonged effect of drug released from SPHs with excellent mechanical properties and the derived polynomial equations were also found to be helpful in predicting the values of selected independent variables for preparation of optimum SPH formulations with desired properties. The SPHs were of low porous nature and having good swelling capacity. The SPHs were observed to be low dense and the higher void fraction of SPHs was associated with decreased concentration of the polymers and cross linker. The SPHs were successfully formulated with HPMC as a gelling agent to sustain the action of the mefenamic acid by improving drug solubility and by imparting viscosity to SPHs. The prolonged action of the drug was made possible by increasing gastric residence time of drug and by enhancing the mechanical properties of SPHs which were achieved through inter-penetrating polymeric network of acrylic acid with HPMC using glycerol as a cross linker. The formulated SPHs showed a significantly rapid, higher and prolonged analgesic activity as compared to that of simple suspension of MA and it could be related with slow release and enhanced absorption of MA released from SPHs. All these characteristics of SPHs would increase the feasibility of using MA loaded SPHs as oral sustained release systems which would be helpful in improving the patients’ compliance. However, further studies are recommended to prove the *in vivo* therapeutic efficacy of MA loaded SPHs by performing detailed pharmacokinetic and pharmacodynamics studies in healthy humans.

## Data Availability

The original contributions presented in the study are included in the article/supplementary material, further inquiries can be directed to the corresponding authors.
